# Neonatal Seizure Management: Is the Timing of Treatment Critical?

**DOI:** 10.1016/j.jpeds.2021.09.058

**Published:** 2022-04

**Authors:** Andreea M. Pavel, Janet M. Rennie, Linda S. de Vries, Mats Blennow, Adrienne Foran, Divyen K. Shah, Ronit M. Pressler, Olga Kapellou, Eugene M. Dempsey, Sean R. Mathieson, Elena Pavlidis, Lauren C. Weeke, Vicki Livingstone, Deirdre M. Murray, William P. Marnane, Geraldine B. Boylan

**Affiliations:** 1 INFANT Research Centre, Cork, Ireland; 2 Department of Pediatrics and Child Health, University College Cork, Cork, Ireland; 3 Institute for Women's Health, University College London, London, United Kingdom; 4 Utrecht Brain Center, University Medical Center Utrecht, Utrecht, the Netherlands; 5 Department of Neonatal Medicine, Karolinska University Hospital, Stockholm, Sweden; 6 Division of Pediatrics, Department of Clinical Science, Intervention, and Technology, Karolinska Institutet, Stockholm, Sweden; 7 Department of Neonatal Medicine, Rotunda Hospital, Dublin, Ireland; 8 Department of Neonatology, Royal London Hospital, London, United Kingdom; 9 The London School of Medicine and Dentistry, Blizard Institute, Queen Mary University of London, London, United Kingdom; 10 Department of Clinical Neurophysiology, Great Ormond Street Hospital for Children NHS Trust, London, United Kingdom; 11 Department of Neonatology, Homerton University Hospital NHS Foundation Trust, London, United Kingdom; 12 Department of Electrical & Electronic Engineering, School of Engineering, University College Cork, Cork, Ireland

**Keywords:** antiseizure medication, newborn, encephalopathy, seizures, aEEG, Amplitude-integrated electroencephalography, cEEG, Conventional electroencephalography, EEG, Electroencephalography, HIE, Hypoxic ischemic encephalopathy

## Abstract

**Objective:**

To assess the impact of the time to treatment of the first electrographic seizure on subsequent seizure burden and describe overall seizure management in a large neonatal cohort.

**Study design:**

Newborns (36-44 weeks of gestation) requiring electroencephalographic (EEG) monitoring recruited to 2 multicenter European studies were included. Infants who received antiseizure medication exclusively after electrographic seizure onset were grouped based on the time to treatment of the first seizure: antiseizure medication within 1 hour, between 1 and 2 hours, and after 2 hours. Outcomes measured were seizure burden, maximum seizure burden, status epilepticus, number of seizures, and antiseizure medication dose over the first 24 hours after seizure onset.

**Results:**

Out of 472 newborns recruited, 154 (32.6%) had confirmed electrographic seizures. Sixty-nine infants received antiseizure medication exclusively after the onset of electrographic seizure, including 21 infants within 1 hour of seizure onset, 15 between 1 and 2 hours after seizure onset, and 33 at >2 hours after seizure onset. Significantly lower seizure burden and fewer seizures were noted in the infants treated with antiseizure medication within 1 hour of seizure onset (*P* = .029 and .035, respectively). Overall, 258 of 472 infants (54.7%) received antiseizure medication during the study period, of whom 40 without electrographic seizures received treatment exclusively during EEG monitoring and 11 with electrographic seizures received no treatment.

**Conclusions:**

Treatment of neonatal seizures may be time-critical, but more research is needed to confirm this. Improvements in neonatal seizure diagnosis and treatment are also needed.

See editorial, p 7

Owing to the unique physiologic properties of the immature brain,^1^ seizures are common in newborn infants, with an incidence of 1-3.5/1000 live births in term infants.^2^, ^3^, ^4^ Although a wide variety of causes have been reported, the leading cause remains hypoxic ischemic encephalopathy (HIE), despite the introduction of therapeutic hypothermia.^3^^,^^5^ Seizure recognition is challenging in newborns because many seizures are subclinical or have subtle clinical manifestations; in addition, treatment of seizures can cause an “uncoupling” of clinical and electroencephalography (EEG) features.^6^

There has been an increase in the use of EEG in neonatal units for diagnosing seizures.^5^^,^^7^ Amplitude-integrated EEG (aEEG) is still preferred by some neonatologists, because it is easy to perform and not dependent on 24/7 neurophysiology support.^8^^,^^9^ International guidelines recommend the use of continuous conventional electroencephalography (cEEG) for a minimum of 24 hours as the gold standard for seizure diagnosis in the newborn.^10^, ^11^, ^12^

There is increasing evidence that neonatal seizures are associated with poor neurodevelopmental outcome, and that untreated seizures might add to the initial brain injury.^13^^,^^14^ Responsiveness to antiseizure medication may decrease with recurrent and prolonged seizures.^15^, ^16^, ^17^ The hypothesis that earlier treatment leads to better response is supported by animal work in mice and rats which has shown a progressive increase in intracellular chloride with recurrent seizures, increasing the likelihood of more seizures and decreasing the responsiveness to treatment.^17^ Current published guidelines for management of all neonatal seizures recommend initiation of antiseizure medication as soon as possible following seizure recognition, but there are no recommendations on specific target times for treatment.^18^ There remains significant variability in how seizures are diagnosed and managed, and a consensus is needed.^19^, ^20^, ^21^

Our group previously showed that only 11% of “seizure episodes” were treated within the first hour after onset.^3^ Another study investigating the time from onset of aEEG seizure to treatment with antiseizure medication found that 32.1% of patients were treated within 1 hour of onset, 19.8% were treated within 1-2 hours of onset, and the majority (48.1%) were treated at >2 hours after onset.^22^ The primary aim of our present analysis was to assess whether the time to treatment of first electrographic seizure has an impact on subsequent seizure burden. To achieve this goal, we used a large multicenter European neonatal cohort. Our secondary aim was to describe the initial seizure management for all infants included in this cohort.

## Methods

The present study is a secondary data analysis of 2 European multicenter cohort studies that recruited newborns across 8 European tertiary neonatal intensive care units between January 2011 and February 2017 (ClinicalTrials.gov identifiers NCT02160171 and NCT02431780). Both studies examined the feasibility and efficacy of cEEG monitoring and a new automated neonatal seizure detection algorithm (ANSeR algorithm)^3^^,^^23^ and included infants of 36-44 weeks corrected gestational age requiring EEG monitoring for suspected seizures. Infants were excluded only if parental/guardian written consent was refused or they were at corrected gestational age <36 weeks. Relevant information regarding delivery and neonatal course were recorded in study-designed electronic databases. To assess the effect of treatment timing of the first electrographic seizure (main aim), we included all infants who received antiseizure medication exclusively after electrographic seizure onset and who had at least 24 hours of cEEG recording after electrographic seizure onset. The secondary aim was to describe the initiation of seizure management for all infants included in the 2 studies.

Ethical approval was granted for both studies by national and local Ethics Committees specific to each participating center.

### EEG Monitoring

All infants underwent prolonged video cEEG monitoring as clinically indicated, using a 10:20 EEG electrode modified neonatal system with disposable electrodes placed at F3, F4, C3, C4, Cz, T3, T4, O1/P3, and O2/P4. Three different EEG machines were used for monitoring: Neurofax EEG-1200 (Nihon Kohden), NicoletOne ICU Monitor (Natus), and XLTek EEG (Natus). Teams at each site were trained in EEG electrode application and maintenance of good quality recordings. The clinical teams at each site had the aEEG signals from F3-C3 and F4-C4, 8 raw EEG channels, and electrocardiography and respiratory traces displayed on the EEG monitors and available to review. No standard EEG review protocol was imposed during the study period, and the clinical teams reviewed the monitoring data as recommended by the local guidelines.

### Seizure Analysis

This analysis included all the EEG monitoring performed for each infant, regardless of the time of study enrollment. Electrographic seizures were annotated by 1 of 4 neonatal neurophysiologists blinded to the infant's medical history and outcome. A standard EEG review protocol for seizure annotation was used. Electrographic seizures were defined as a minimum 10 seconds of sudden, repetitive, and evolving stereotyped waveforms involving at least 1 EEG channel.^24^ An infant was considered to have seizures if at least 1 electrographic seizure was annotated. The following summary measures of seizures were calculated: seizure period (time [in hours] from the beginning of the first electrographic seizure to the end of the last electrographic seizure), total seizure burden (duration [in minutes] of all seizures occurring during the entire monitoring period), maximum hourly seizure burden (maximum seizure burden within 1 hour [in minutes/hour]), status epilepticus (seizure burden in a minimum of 30 minutes within 1 hour), and number of seizures during the entire monitoring period. Seizure characteristics were described for all infants who experienced seizures during the EEG monitoring period.

### Antiseizure Medication Treatment

EEG recordings were reviewed by local teams from each site in real time, and all seizures detected were managed according to local protocols. For each infant, antiseizure medication treatment was recorded in the study database (ie, type of drug, dose, and time of administration). The antiseizure medication was chosen according to local protocols and at the clinician's discretion.

### Treatment Timing Analysis

For the purpose of treatment timing analysis, we considered the first antiseizure medication dose administered after the first electrographic seizure. This analysis included only infants with antiseizure medication given exclusively after electrographic seizure onset and with a minumum of 24 hours of EEG. Infants who received antiseizure medication before their first electrographic seizure and infants who did not receive antiseizure medication treatment throughout the study period were excluded from this analysis, as this could be a bias for seizure diagnosis and management by the clinical teams. The treatment timing cohort was divided into 3 groups: treatment with antiseizure medication within 1 hour of electrographic seizure onset, between 1 and 2 hours after electrographic seizure onset, and at >2 hours after electrographic seizure onset. The primary outcome was seizure burden, and the secondary outcomes were maximum seizure burden, presence of status epilepticus, number of seizures, and total number of antiseizure medication doses. The American Clinical Neurophysiology Society guidelines recommend at least 24 hours of cEEG monitoring for neonates at risk of seizures and when seizures are confirmed, at least 24 hours of seizure-free cEEG^10^; therefore, all outcomes were calculated over the first 24 hours after electrographic seizure onset. A post hoc analysis was performed in the subgroup of infants diagnosed with HIE, from which infants with encephalopathy caused by factors other than HIE injury were excluded.

### Statistical Analyses

Data are reported as median and IQR for continuous variables and as frequency and percentage for categorical variables. Differences in outcomes between the treatment groups were investigated based on type of data and normality. The Mann-Whitney U test and Kruskal-Wallis test were used for continuous outcome variables, and the χ^2^ test was used for categorical outcome variables. Post hoc pairwise comparisons with a Bonferroni correction were performed if the omnibus test was significant. All tests were 2-sided, and a *P* value < .05 was considered statistically significant. Statistical analysis was performed using SPSS version 25.0 (IBM).

## Results

**Figure 1:**
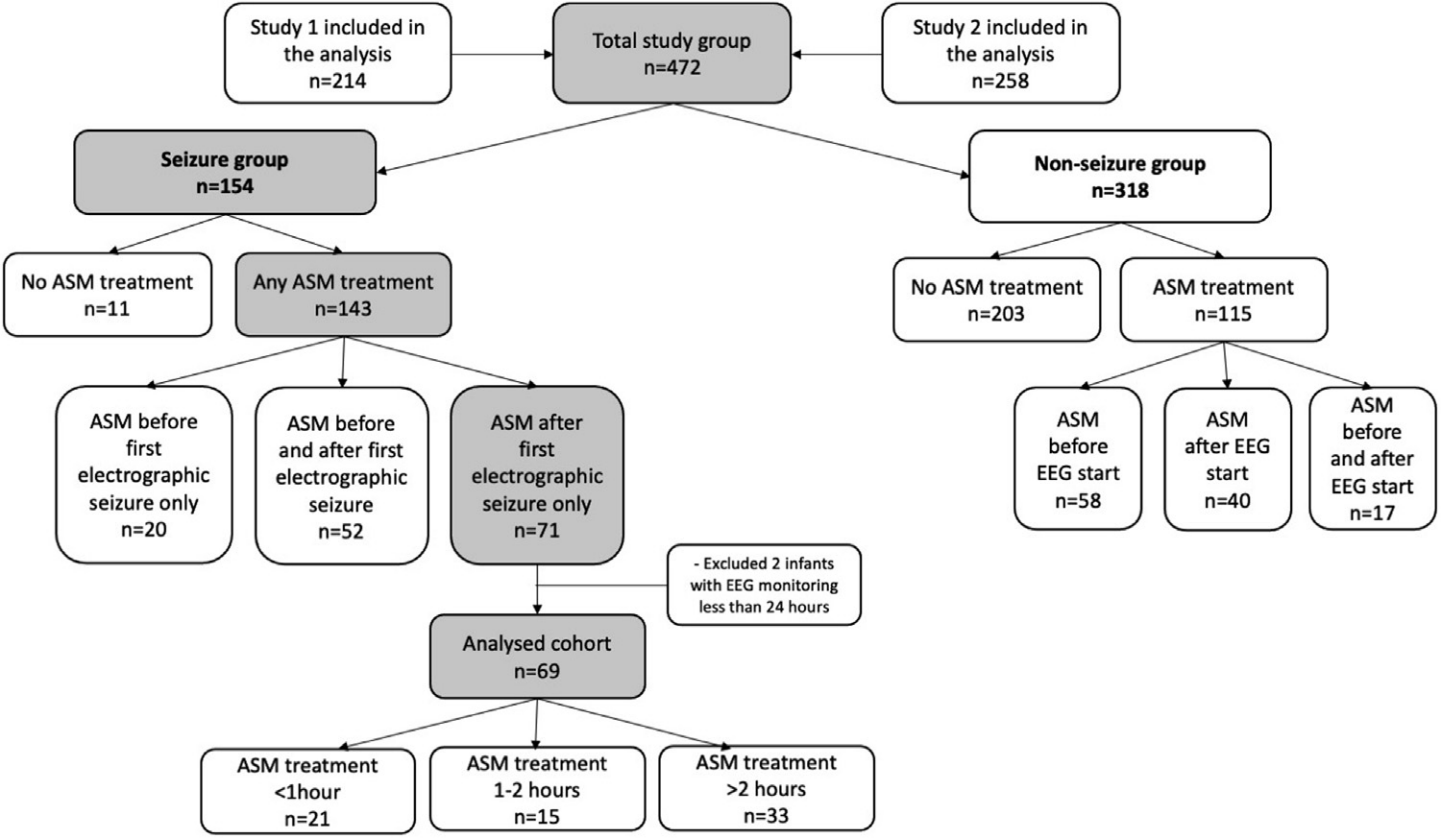
Study flow diagram. *ASM,* anti-seizure medication; *EEG,* electroencephalography.

The 2 studies included a total of 472 infants: 318 (67.4%) without seizures and 154 (32.6%) with seizures (Figure 1). The neonatal characteristics for the whole cohort are presented in Table I (available at www.jpeds.com). The percentages of infants with moderate and severe HIE, stroke, and metabolic/genetic disorders were higher in the seizure group compared with nonseizure group, but otherwise the 2 groups were similar.

### Seizure Characteristics and Treatment

Out of 154 infants with evidence of electrographic seizures, 31 (20.1%) had no antiseizure medication given after electrographic seizure onset, including 11 who received no antiseizure medication at all and 20 who received antiseizure medication only before electrographic seizure onset (Table II). Based on the timing of antiseizure medication administration after electrographic seizure onset, 26 infants (16.9%) received treatment within 1 hour, 23 (14.9%) received treatment between 1 and 2 hours, and 74 (48.1%) received treatment after 2 hours.

**Table II:** Seizure characteristics∗ of all infants with electrographic seizures and for infants by treatment timing group after electrographic seizure onset: descriptive analysis

Characteristics	All (n = 154)	Antiseizure medication treatment at <1 h (n = 26)	Antiseizure medication treatment at 1-2 h (n = 23)	Antiseizure medication treatment at >2 h (n = 74)	No antiseizure medication treatment (n = 31)
General characteristics					
Seizure period, h, median (IQR)	16.5 (6.7-40.2)	18.1 (0.7-57.4)	16.6 (8.3-54.5)	26.6 (10.0-48.2)	8.1 (2.7-14.8)
Total seizure burden, min, median (IQR)	69 (23-154)	45 (21-127)	104 (34-167)	75 (27-162)	30 (4-106)
Number of seizures, median (IQR)	21 (9-52)	23 (5-33)	28 (12-52)	32 (12-60)	10 (2-32)
Median seizure duration, s, median (IQR)	104 (65-189)	89 (56-495)	105 (75-191)	108 (64-160)	98 (45-164)
Maximum seizure burden, min/h, median (IQR)	22 (10-32)	22 (12-36)	24 (16-35)	22 (10-31)	14 (2-28)
Age when maximum seizure burden was reached, h, median (IQR)	35 (19-63)	21 (11-49)	32 (19-80)	36 (20-68)	38 (28-59)
Status epilepticus (yes), n (%)	43 (27.9)	8 (30.8)	8 (34.8)	20 (27.0)	7 (22.6)
Characteristics related to first electrographic seizure					
Age at first electrographic seizure, h, median (IQR)	22 (14-54)	14 (9-39)	20 (15-57)	21 (14-56)	36 (19-59)
Duration of first seizure, s, median (IQR)	93 (46-273)	268 (65-1385)	104 (37-681)	75 (43-161)	114 (45-287)
Seizure burden in the first hour of seizure period, min, median (IQR)	6.0 (2.3-15.1)	15.7 (10.0-30.8)	9.7 (2.6-22.1)	4.4 (1.8-10.2)	3.9 (1.9-11.7)
Number of seizures in the first hour of seizure period, median (IQR)	2 (1-4)	2 (1-6)	3 (1-4)	2 (1-4)	2 (1-3)

∗ Seizure characteristics were calculated based on complete EEG monitoring throughout the study period.

The median age of seizure onset for all infants was 22 (IQR, 14-54) hours after birth, with a median total seizure burden of 69 (IQR, 23-154) minutes. Compared with infants in the other groups, those in the group treated with antiseizure medication within 1 hour had an earlier onset of seizure, a longer duration of first electrographic seizure, and a higher seizure burden in the first hour after seizure onset (Table II).

### Primary Aim: Treatment Timing Analysis

This analysis included infants who received antiseizure medication exclusively at any time point after the first electrographic seizure (n = 69 infants) (Table III). We excluded infants who did not receive any antiseizure medication during the study period (n = 11), infants who received antiseizure medication exclusively before electrographic seizure onset (n = 20), infants who received antiseizure medication both before and after electrographic seizure onset (n = 52), and infants with <24 hours of EEG monitoring after their first electrographic seizure (n = 2).

**Table III:** Antiseizure treatment group analysis (n = 69)

Outcomes	Groups based on antiseizure medication treatment timing for first electrographic seizure			*P* value∗	Pairwise comparison
	Treatment at <1 h (n = 21)	Treatment at 1-2 h (n = 15)	Treatment at >2 h (n = 33)		
Seizure burden within 24 h, min, median (IQR)	36 (15-70)	71 (32-112)	75 (30-152)	.029	<1 h vs >2 h
Maximum seizure burden within 24 h, min/h, median (IQR)	16 (11-24)	20 (12-40)	27 (12-35)	.224	
Number of seizures within 24 h, median (IQR)	10 (2-24)	18 (6-32)	28 (11-50)	.035	<1 h vs >2 h
Status epilepticus within 24 h (yes), n (%)	3 (14.3)	4 (26.7)	14 (42.4)	.089†	
Total doses of antiseizure medication within 24 h, median (IQR)	2 (1-4)	2 (1-3)	2 (1-3)	.712	

The infants included in this analysis were those who received no antiseizure medication before their first electrographic seizure, with at least 1 antiseizure medication dose given after the first electrographic seizure and with at least 24 hours of EEG monitoring after the first electrographic seizure.

∗ *P* values were from the Kruskal-Wallis test unless indicated otherwise. *P* < .05 was considered to indicate statistical significance.

† *P* value from the χ^2^ test.

**Figure 2:**
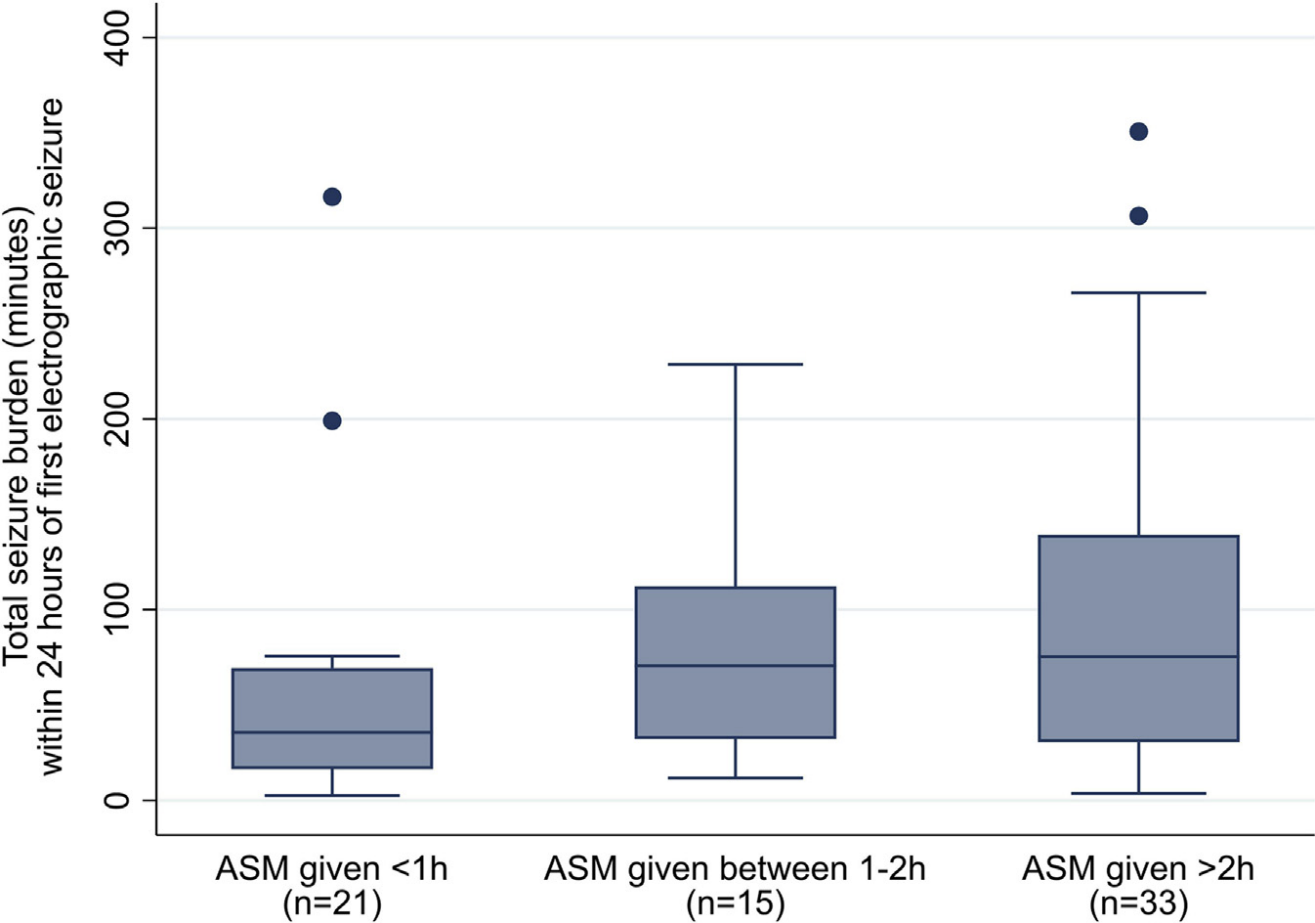
Seizure burden within 24 hours from electrographic seizure onset by treatment groups (boxplot). *ASM,* anti-seizure medication.

Seizure burden and the number of seizures differed significantly between the antiseizure medication treatment groups (*P* = .029 and .035, respectively). The pairwise analysis showed that from the onset of first electrographic seizure, the seizure burden calculated over the subsequent 24 hours was significantly lower in the <1 hour antiseizure medication group compared with the >2 hour antiseizure medication group (median seizure burden 36 [IQR, 15-70] minutes vs 75 [IQR, 30-152] minutes; *P* = .025) (Figure 2; available at www.jpeds.com). The number of seizures was also significantly lower in the <1 hour group compared with the >2 hour group (median number of seizures, 10 [IQR, 2-24] vs 28 [IQR, 11-50]; *P* = .032).

We investigated etiology, therapeutic hypothermia status, age at start of EEG monitoring, and age at first electrographic seizure as potential confounding variables and found that they were not associated with the timing of antiseizure medication administration. The background etiologies were not statistically significantly different among the antiseizure medication treatment groups (<1 hour group: moderate HIE, n = 7 [33.3%]; severe HIE, n = 5 [23.8%]; stroke, n = 3 [14.3%]; other, n = 6 [28.6%]; 1-2 hour group: moderate HIE, n = 5 [33.3%]; severe HIE, n = 5 [33.3%]; stroke, n = 3 [20%]; other, n = 2 [13.3%]; >2 hours group: moderate HIE, n = 12 [36.4%]; severe HIE, n = 8 [24.2%]; stroke, n = 7 [21.2%]; other, n = 6 [18.2%]; *P* = .928). Therapeutic hypothermia was provided to 12 infants (57.1%) in the <1 hour group, 9 infants (60%) in the 1-2 hours group, and 18 infants (54.5%) in the >2 hours group (*P* = .937). The median age at the start of EEG monitoring 6.9 (IQR, 3.4-38.6) hours of life in the <1 hour group, 9.0 (IQR, 3.0-41.6) hours of life in the 1-2 hours group, and 6.9 (IQR, 3.6-33.1) hours of life in the >2 hours group (*P* = .976). The median age at the first electrographic seizure was 14.1 (IQR, 8.6-39.6) hours of life in the <1 hour group, 16.2 (IQR, 9-42.3) hours of life in the 1-2 hours group, and 14.6 (9.9-48.2) hours of life in the >2 hours group (*P* = .851).

Comparing the group of infants who received antiseizure medication exclusively after their first electrographic seizure (n = 69) with the group who received antiseizure medication before and after their first seizure (n = 52), there were no significant differences in terms of total seizure burden (median, 74 [IQR, 32-167] minutes vs 79 [IQR, 21-158] minutes; *P* = .582), maximum seizure burden (median, 24 [IQR, 13-34] minutes vs 19 [IQR, 8-29] minutes; *P* = .071), number of seizures (median, 25 [IQR, 10-50] vs 33 [IQR, 12-58]; *P* = .449), and presence of status epilepticus (24 [34.8%] vs 12 [23.1%]; *P* = .163).

Post hoc analysis also was performed in the subgroup of infants who had a diagnosis of HIE (n = 42), and the seizure burden was significantly different among the antiseizure medication treatment groups (*P* = .009). The pairwise comparison showed a significantly lower seizure burden in the <1 hour group compared with the >2 hours group (median 41 [IQR, 24-67] minutes vs 86 [IQR, 68-168] minutes; *P* = .007) (Table IV). For the HIE cohort, therapeutic hypothermia status and age at the start of EEG monitoring were investigated as potential confounders and were not associated with antiseizure medication timing (*P* = .834 and = .984, respectively).

**Table IV:** Antiseizure treatment group analysis for infants with HIE (n = 42)

Outcomes	Groups based on antiseizure medication treatment timing for first electrographic seizure			*P* value∗	Pairwise comparison
	Treatment at <1 h (n = 12)	Treatment at 1-2 h (n = 10)	Treatment at >2 h (n = 20)		
Seizure burden within 24 h, min, median (IQR)	41 (24-67)	87 (34-113)	86 (68-168)	.009	<1 h vs >2 h
Maximum seizure burden within 24 h, min/h, median (IQR)	21 (13-35)	24 (11-41)	32 (23-40)	.132	
Number of seizures within 24 h, median (IQR)	10 (1-23)	15 (6-34)	24 (11-58)	.056	
Status epilepticus within 24 h (yes), n (%)	3 (25.0)	3 (30.0)	12 (60.0)	.098†	
Total doses of antiseizure medication within 24 h, median (IQR)	2 (1-4)	3 (2-3)	2 (1-4)	.909	

The infants included in this analysis were those who received no antiseizure medication before their first electrographic seizure, with at least 1 antiseizure medication dose given after the first electrographic seizure and with at least 24 hours of EEG monitoring after the first electrographic seizure.

∗ *P* values were from the Kruskal-Wallis test unless indicated otherwise. *P* < .05 was considered to indicate statistical significance.

† *P* value from the χ^2^ test.

### Secondary Aim: Overall Description of Antiseizure Medication Treatment

Among the 472 infants, 258 (54.7%) received at least 1 dose of antiseizure medication before or during EEG monitoring. In the nonseizure group, 115 infants (36.2%) received at least 1 dose of antiseizure medication, including 58 infants before the start of EEG monitoring, 40 infants during EEG monitoring, and 17 infants before and during EEG monitoring. Forty-seven infants with no electrographic seizures received multiple antiseizure medication doses throughout the study period. In the seizure group, 143 infants (92.9%) received at least 1 dose of antiseizure medication; however of those, 20 infants (14.0%) received antiseizure medication exclusively before the onset of electrographic seizures (including the period before start of EEG monitoring).

Out of 258 infants treated for seizures, phenobarbital was the most common first line treatment of choice (90.7% of infants), followed by Midazolam (7.0%), Lignocaine (0.8%), Levetiracetam (0.4%), Lorazepam (0.4%), Biotin (0.4%) and Paraldehyde (0.4%).

## Discussion

The findings of this study demonstrate that the group of infants with electrographic seizures treated within 1 hour of seizure onset had the lowest seizure burden and fewer seizures over the subsequent following 24 hours (despite a higher seizure burden in the first hour in this group) compared with infants who received antiseizure medication after 1 hour of seizure onset. Similarly, the post hoc analysis investigating treatment timing in infants with a diagnosis of HIE showed a lower seizure burden in the early (within 1 hour) treatment group.

Current international guidelines recommend that treatment for neonatal seizures should be administered as soon as possible but do not specify an optimal treatment target time.^18^ The ANSeR phase 1 study cohort reported by Rennie et al was included in this analysis, together with a neonatal cohort (ANSeR phase 2 study) recruited for a randomized controlled trial of a seizure detection algorithm.^3^^,^^23^ Rennie et al showed that only 11% of “seizure episodes” (clusters of seizures separated by <2 hours) were treated within 1 hour. In the present study, we analyzed only the treatment of first electrographic seizures and found that among all the infants with electrographic seizures, 26 (16.9%) received treatment for their first electrographic seizure within the first hour; however, only 21 received antiseizure medication exclusively after the onset of electrographic seizure (5 infants received antiseizure medication before the start of EEG monitoring due to clinical seizures). In another study, 32.1% of the infants received antiseizure medication starting within 1 hour of aEEG seizure onset.^22^ The higher proportion of early treatment in that study compared with our cohort could be explained by their use of a seizure detection algorithm and a single expert site. Previous studies have shown that a high seizure burden is independently associated with worse brain injury detected on magnetic resonance imaging and worse neurodevelopmental outcomes, suggesting that early antiseizure medication administration resulting in a reduction of seizure burden might lead to a decrease in magnetic resonance imaging–detected brain injury and better long-term outcomes.^13^^,^^25^, ^26^, ^27^ A randomized controlled trial demonstrated an increased efficacy of phenobarbital for neonatal seizures, which could be related to earlier treatment facilitated by a frequent review of the EEG monitoring and rapid diagnosis of electrographic seizures.^28^ Although we cannot make a direct comparison with the previous study, our present findings show a lower seizure burden in infants treated within 1 hour of electrographic seizure onset, suggesting a possible association between antiseizure medication timing and seizures. Furthermore, the seizure burden was similar if antiseizure medication was received after the 1-hour cutoff (the antiseizure medication at 1-2 hours and >2 hours groups), suggesting that the impact of antiseizure medication on seizure burden might be optimal if administered within 1 hour of seizure onset.

We evaluated the different etiologies in our cohort, therapeutic hypothermia status, age at initiation of EEG monitoring, and age at first electrographic seizure as possible confounders for this analysis but noted no significant differences among the treatment groups. Our results are supported by previous animal and human work demonstrating that the sooner the treatment, the better the response.^17^^,^^29^

For our analysis of treatment timing, we had a large multicenter European neonatal cohort from eight tertiary neonatal intensive care units available, and thus we also described the overall seizure management in this cohort. Out of 472 newborns, 258 (54.7%) received at least 1 dose of antiseizure medication during the study period. Seventy-eight infants received antiseizure medication before EEG monitoring was started for suspected clinical seizures, among whom 58 infants had no evidence of further electrographic seizures. We can only assume that in these infants, the seizures responded to the initial antiseizure medication treatment, or that what was diagnosed as a clinical seizure was likely part of a neonatal movement disorder rather than a true seizure, as demonstrated previously by our group.^30^

The majority of infants who received antiseizure medication exclusively during EEG monitoring had demonstrated electrographic seizures; however, 40 infants received antiseizure medication exclusively during EEG monitoring and had no evidence of electrographic seizures. Most of the infants who did not receive antiseizure medication had no electrographic seizures throughout the duration of EEG monitoring (203 infants); however, 11 infants with electrographic seizures had no treatment. Among these 11 infants with electrographic seizures, 6 had a total seizure burden of >20 minutes and 2 had status epilepticus. These results demonstrate that recognition of seizures remains a major challenge for neonatologists and inappropriate antiseizure medication treatment (undertreatment and overtreatment) remains an ongoing concern. Animal and human studies have documented that exposure to antiseizure medication can lead to neuronal apoptosis, poor brain development, and later cognitive impairments; therefore, it is important to administer antiseizure medication appropriately to infants who may already have some degree of brain injury.^31^, ^32^, ^33^, ^34^, ^35^ These results illustrate the diagnostic difficulties that clinicians face, even when the gold standard diagnostic tool (cEEG) is used. Current guidelines recommend the use of cEEG monitoring for at least 24 hours to detect seizures in neonates, but there are no clear protocols for EEG review, and neurophysiologic support is limited even in tertiary neonatal centers.^10^ Implementation of EEG monitoring and seizure management protocols and specific EEG review and interpretation training have been shown to be beneficial.^29^^,^^36^

Several limitations must be considered when interpreting our present results. This is a secondary analysis of infants recruited to 2 European studies of cEEG monitoring, not a prospective study investigating treatment of neonatal seizures. Given the sample sizes, only large differences between antiseizure medication groups could be detected across the outcomes investigated. When seizures were detected, treatment was initiated in accordance with local clinical guidelines, and because there is no consensus on the optimal target time for treatment, the treatment cutoffs used for this analysis were selected based on previous literature.^3^^,^^22^ This analysis included electrographic seizures with a minimum duration of 10 seconds, which might be considered by some as too short to intervene. However only 14 of 154 infants had a first seizure of <30 seconds duration, and only 1 of these infants was included in the treatment timing analysis. Excluding this infant from the analysis did not change the significance of the results. On the other hand, the most recent International League Against Epilepsy definition of neonatal seizure does not include a minimum duration of 10 seconds as long as an evolution in frequency and morphology is demonstrated.^37^ In our cohort, the mean duration of the first electrographic seizure was 93 (IQR, 46-273) seconds, and only 9% of the infants had a first electrographic seizure of <30 seconds. Although we cannot definitely rule out that this did not influence the overall results, we believe that it is unlikely, given that the majority of seizures in our cohort were >10 seconds. However, we do believe that a more detailed analysis of shorter-duration discharges using the International League Against Epilepsy definition could provide valuable insight into the impact of electrographic seizures on the developing brain and is a priority area for our future research.

For our treatment timing analysis, infants who received antiseizure medication before electrographic seizure onset (including previous EEG start) were excluded to minimize the cumulative effect of antiseizure medication on the electrographic seizure burden. When comparing the group of infants with antiseizure medication given exclusively after the first electrographic seizure (n = 69 infants) with the group of infants with antiseizure medication given before and after the first electrographic seizure (n = 52 infants), we found no significant differences in terms of total seizure burden, maximum seizure burden, number of seizures, or presence of status epilepticus. In addition, infants who did not receive antiseizure medication after the emergence of electrographic seizure were excluded, because electrographic seizures were not recognized and not treated by the cotside clinical teams. As a result, we could include only 45% (69 of 154) of all infants with electrographic seizures in the treatment timing analysis. Comparing the group of infants included in the treatment timing analysis (n = 69) with the group of infants excluded (n = 85), we noted that more than one-half of the excluded infants were born outside of the recruiting hospitals, and thus the EEG monitoring was started significantly later in this group. All participating centers were tertiary referral hospitals, and almost one-half of the infants with electrographic seizures were outborn. This may explain the high proportion of infants who received antiseizure medication before EEG commencement.

This analysis looked at short-term outcomes, derived from the seizure burden, and our 2-year developmental follow-up analysis is not yet completed. Our findings suggest that there may be an association between treatment timing and subsequent seizure burden and that treatment of neonatal seizures might be time-critical; however, this needs to be confirmed in a large prospective study. Inappropriate treatment remains an ongoing concern; many infants continue to be treated who do not require treatment and in those who do, delayed onset of treatment remains problematic. Recognition of electrographic seizures remains a major challenge for neonatologists. With the increasing use of prolonged neonatal cEEG monitoring and insufficient neonatal neurophysiology expertise, additional support from automated seizure detection algorithms might be the solution.^23^^,^^38^^,^^39^

## Data Statement

Data sharing statement available at www.jpeds.com.

## Figures and Tables

**Table I:** Neonatal characteristics

Characteristics	All infants		Seizure group		Non-seizure group		*P* value∗
	Number	Value	Number	Value	Number	Value	
Gestational age at birth, wk, median (IQR)	472	40 (39-41)	154	40 (39-41)	318	40 (38-41)	.117
Birth weight, g, median (IQR)	472	3440 (3061-3800)	154	3400 (3088-3793)	318	3450 (3058-3818)	.954
Male sex, n (%)	472	292 (61.9)	154	91 (59.1)	318	201 (63.2)	.388
Mode of delivery, n (%)	470		154		316		.372
Spontaneous vaginal delivery		171 (36.4)		57 (37.0)		114 (36.1)	
Instrumental vaginal delivery		126 (26.8)		35 (22.7)		91 (28.8)	
Elective cesarean delivery		43 (9.1)		18 (11.7)		25 (8.0)	
Emergency cesarean delivery		130 (27.7)		44 (28.6)		86 (27.1)	
Place of birth, n (%)	472		154		318		.648
Inborn in recruiting hospital		255 (54.0)		81 (52.6)		174 (54.7)	
Outborn home/prehospital		19 (4.0)		8 (5.2)		11 (3.5)	
Outborn other hospital		198 (42.0)		65 (42.2)		133 (41.8)	
Apgar score at 1 min, median (IQR)	447	3 (1-7)	148	2 (1-8)	299	3 (1-7)	.094
Apgar score at 5 min, median (IQR)	451	6 (4-9)	148	6 (3-9)	303	6 (4-9)	.185
Assisted ventilation at 10 min of life (yes), n (%)	461	216 (46.9)	153	76 (49.7)	308	140 (45.5)	.393
Lowest cord pH, median (IQR)	339	7.11 (6.93-7.23)	104	7.10 (6.90-7.20)	235	7.12 (6.94-7.24)	.182
Therapeutic hypothermia (yes), n (%)	472	234 (49.6)	154	84 (54.5)	318	150 (47.2)	.133
Diagnosis before discharge, n (%)	472		154		318		<.001
Mild HIE		82 (17.4)		0 (0)		82 (26.0)	
Moderate HIE		125 (26.5)		50 (32.5)		75 (23.6)	
Severe HIE		56 (11.9)		40 (26.0)		16 (5.0)	
Stroke		50 (10.5)		26 (16.9)		24 (7.5)	
Metabolic/genetic disorder		43 (9.1)		27 (17.5)		16 (5.0)	
Suspected seizures, unconfirmed		31 (6.6)		0 (0)		31 (9.7)	
Perinatal asphyxia without encephalopathy		22 (4.7)		0 (0)		22 (7.0)	
Sepsis/meningitis		20 (4.2)		3 (2.0)		17 (5.3)	
Intracranial hemorrhage		10 (2.1)		1 (0.6)		9 (2.8)	
Other†		33 (7.0)		7 (4.5)		26 (8.2)	
Age at start of EEG monitoring, h, median (IQR)	472	14.5 (5.6-44.4)	154	14.5 (5.7-41.0)	318	14.5 (5.5-49.1)	.576
Duration of EEG monitoring, h, median (IQR)	472	65.7 (29.6-92.8)	154	86.4 (52.2-102.6)	318	47.6 (23.5-88.0)	<.001

∗ *P* values were from the Mann-Whitney *U* test for continuous data and the χ^2^ test for categorical data unless indicated otherwise. *P* < .05 was considered to indicate statistical significance.

† n = 8 seizures of unknown origin; n = 7 transient metabolic deficit; n = 5 congenital brain malformation; n = 3 for each of postnatal cardiorespiratory arrest and respiratory distress; n = 2 for each of neonatal drug withdrawal syndrome and congenital cardiac anomaly; n = 1 for each of congenital anemia, meconium aspiration syndrome, tracheoesophageal atresia with cystic periventricular leukomalacia.

## Supplementary Data

Data Statement

STROBE_checklist
