# Supplementary material for: Neonatal Seizure Management: Is the Timing of Treatment Critical?
Source: J Pediatr. 2022 Apr;243:61–68.e2. doi: 10.1016/j.jpeds.2021.09.058 (PMC9067353; doi:10.1016/j.jpeds.2021.09.058)
Supplement: Data Statement [file mmc1.docx]

**Data sharing statement**: It is currently not possible to share the studies datasets. The clinical data were collected under a written proxy consent from the participants’ guardians/parents which did not include permission for sharing or open data. To be allowed to share this data under Irish Health Research Regulations we will require to re-consent or to obtain approval by the Health Regulation Consent Declaration Committee.
